# Boerhaave syndrome as a complication of colonoscopy preparation: a case report

**DOI:** 10.1186/1752-1947-5-544

**Published:** 2011-11-05

**Authors:** Nikos Emmanouilidis, Mark Dietrich Jäger, Michael Winkler, Jürgen Klempnauer

**Affiliations:** 1 Department of General, Visceral and Transplant Surgery, Hannover Medical School, Carl Neuberg Strasse 1, D-30625 Germany

## Abstract

**Introduction:**

Colonoscopy is one of the most frequently performed elective and invasive diagnostic interventions. For every colonoscopy, complete colon preparation is mandatory to provide the best possible endoluminal visibility; for example, the patient has to drink a great volume of a non-resorbable solution to flush out all feces. Despite the known possible nauseating side effects of colonoscopy preparation and despite the knowledge that excessive vomiting can cause rupture of the distal esophagus (Boerhaave syndrome), which is a rare but severe complication with high morbidity and mortality, it is not yet a standard procedure to provide a patient with an anti-emetic medication during a colon preparation process. This is the first report of Boerhaave syndrome induced by colonoscopy preparation, and this case strongly suggests that the prospect of being at risk of a severe complication connected with an elective colonoscopy justifies a non-invasive, inexpensive yet effective precaution such as an anti-emetic co-medication during the colonoscopy preparation process.

**Case presentation:**

A 73-year-old Caucasian woman was scheduled to undergo elective colonoscopy. For the colonoscopy preparation at home she received commercially available bags containing soluble polyethylene glycol powder. No anti-emetic medication was prescribed. After drinking the prepared solution she had to vomit excessively and experienced a sudden and intense pain in her back. An immediate computed tomography (CT) scan revealed a rupture of the distal esophagus (Boerhaave syndrome). After initial conservative treatment by endoluminal sponge vacuum therapy, she was taken to the operating theatre and the longitudinal esophageal rupture was closed by direct suture and gastric fundoplication (Nissen procedure). She recovered completely and was discharged three weeks after the initial event.

**Conclusions:**

To the best of our knowledge, this is the first report of a case of Boerhaave syndrome as a complication of excessive vomiting caused by colonoscopy preparation. The case suggests that patients who are prepared for a colonoscopy by drinking large volumes of fluid should routinely receive an anti-emetic medication during the preparation process, especially when they have a tendency to nausea and vomiting.

## Introduction

Spontaneous esophageal perforation, or Boerhaave syndrome, is a rare but severe complication caused by excessive vomiting. In Hermann Boerhaave's first report (1724) of a spontaneous esophageal rupture, he described the case of a man who deliberately and repeatedly induced vomiting after a rich meal [1]. In contrast to Boerhaave syndrome, which involves a complete rupture of the esophagus, Mallory-Weiss syndrome [2] is characterized by fissure-like lesions of the mucosa, which are characteristically arranged around the circumference of the cardiac opening along the longitudinal axis of the esophagus. Mallory-Weiss lesions extend up into the esophagus or down into the cardiac opening of the stomach and can be perceived as an incomplete Boerhaave syndrome [3]. While Boerhaave syndrome presents with extensive retrosternal and paravertebral back pain, patients with Mallory-Weiss are usually brought to medical attention by violent retching followed by hematemesis [4].

The typical location of a Boerhaave perforation is the left distal esophagus just above the distal esophageal sphincter. Korn *et al*. [5] described a match of the typical location of the Boerhaave rupture with the contact zone of 'clasp' and oblique muscle fibers at the distal esophageal sphincter. This location is often associated with the coexistence of a hiatus hernia and/or a localized loss of elasticity of the esophagus wall due to chronic esophageal alterations such as scarring transformations induced, for example, by gastric reflux, Barrett's lesions or small injuries after repeated episodes of vomiting [1,6-9]. In most reported cases, vomiting was induced by excessive alcohol misuse [8-11] or other forms of intoxication [12]. Very few other causes have been described; they include Boerhaave syndrome caused by gastroscopy [13]. To the best of our knowledge, this is the first report of a case of Boerhaave syndrome as a consequence of colonoscopy preparation.

## Case presentation

A 73-year-old Caucasian woman was scheduled to undergo elective colonoscopy. She had no history of gastric reflux or any other record of an upper gastrointestinal chronic or acute disease. Her known medical history consisted of mild hypertension, a prosthetic hip joint, and colon diverticulosis.

**Figure 1:**
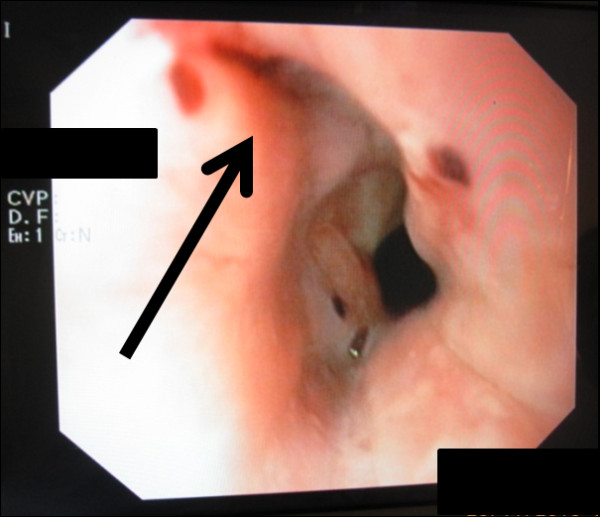
**A Boerhaave perforation was suspected at the site of a small laceration just above the Z-line on the left side of the esophagus**. Other than that, the esophagus and the stomach were not altered.

**Figure 2:**
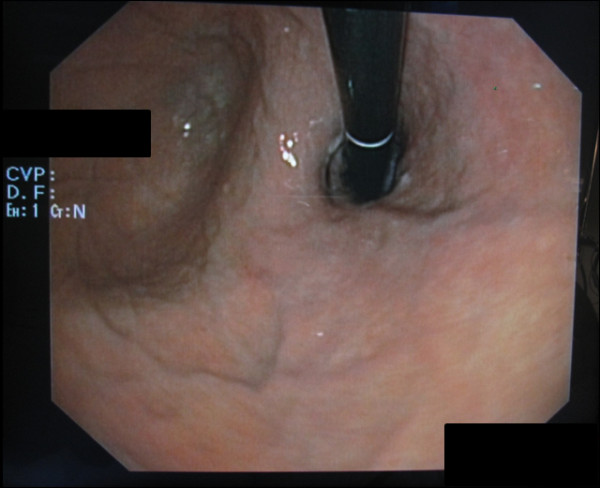
**On inversion there were no visible signs of a perforation of the esophagus from the gastric side**.

**Figure 3:**
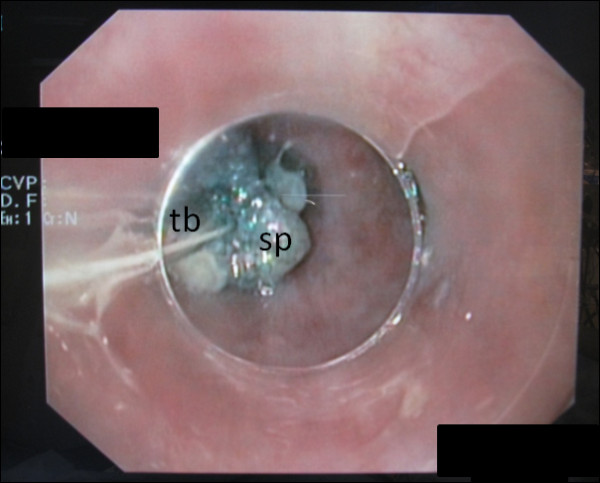
**A small cylinder-shaped polyurethane sponge (sp) of dimensions 10 × 40 mm was sewn to a gastric tube (tb) and placed by endoscopy at the suspected lesion and connected via tb with the VAC therapy unit at 125 mmHg of continuous suction**.

In preparation for the colonoscopy, at home she received soluble MoviPrep powder bags. Anti-emetic medication was not prescribed. MoviPrep is an osmotic laxative whose main component is polyethylene glycol (PEG-3350) and which also contains sodium sulfate, sodium chloride, potassium chloride, sodium ascorbate, ascorbic acid and the additives aspartame, acesulfame-potassium, orange/lemon aroma, maltodextrin and sugar. She followed the manufacturer's instructions and performed the first colon lavage in the afternoon of the day prior to the day of examination by drinking the first 1000 ml of MoviPrep solution in portions of 200 ml ea. as well as drinking the corresponding amount of 1000 mL of water within two hours. The first colon lavage was successful and uneventful. In the early morning on the day of colonoscopy she started the second colon lavage by drinking two portions of 200 mL of PEG solution. and the appropriate additional amount of water. A few minutes later she suddenly became nauseous and was forced to vomit excessively. At the same time she felt a sudden pain in the middle of her back below the left scapula region. Her relatives called the emergency services and she was transferred to the next county hospital. In the emergency room she presented with signs of an acute abdomen (abdomen tender and bloated) and persistent and slightly increasing back pain. An immediate computed tomography (CT) scan with oral contrast medium (CM) was conducted and showed a CM extravasation at the level of the lower thoracic esophagus just above the esophagogastric junction and a small mediastinal emphysema. Boerhaave syndrome was diagnosed and the woman was transferred to the nearby university hospital for further treatment. After transfer, an endoscopic evaluation was conducted and only a small longitudinal laceration (length approximately 15 mm) just above the Z line on the left side of the esophagus was seen and suspected as the site of perforation (Figure 1, arrow). The lesion was not visible when evaluated in inversion from the gastric side (Figure 2). Therefore, we initially considered the perforation to be treatable by endo-sponge vacuum therapy. A polyurethane VAC sponge (V.A.C.^® ^GranuFoam™ Dressing, Kinetic Concepts, Inc. P.O. Box 659508 San Antonio, TX 78265) was placed endoscopically in the esophageal lumen at the height of the lesion and was connected via a small gastric tube (Figure 3) to a VAC therapy unit (ActiV.A.C.^® ^Therapy Unit, Kinetic Concepts, Inc. P.O. Box 659508 San Antonio, TX 78265) with continuous suction at 125 mmHg. She also received a thoracic drain to the left hemithorax. The sponge was left in place for approximately eight hours until a CT scan with oral CM was performed the next morning.

**Figure 4:**
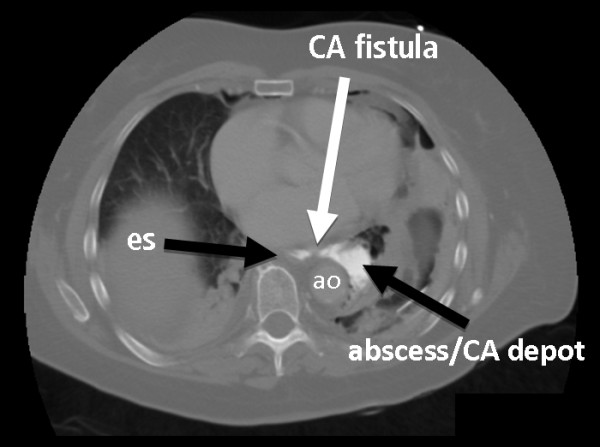
**A computed tomography (CT) scan with oral contrast agent (CA) revealed only a thin CA line between the lumen of the esophagus and the mediastinal paraesophageal abscess/CA depot (es, esophagus; ao, aorta)**.

The CT scan revealed persistent and significant CM leakage with gradual abscess formation and advancing mediastinal emphysema. At the same time, the CT scan showed only a thin filament-like CM fistula between the para-esophageal CM depot and the lumen of the esophagus (Figure 4). Furthermore, clinically our patient slightly deteriorated by developing fever and increasing back pain. Her C-reactive protein and leukocyte levels were also increasing. We assumed that the small longitudinal laceration had a valve-like configuration and thus would not allow the VAC system to have a sufficient abscess draining effect. Thus, we decided to switch from the endoscopic therapeutic approach to open surgery.

**Figure 5:**
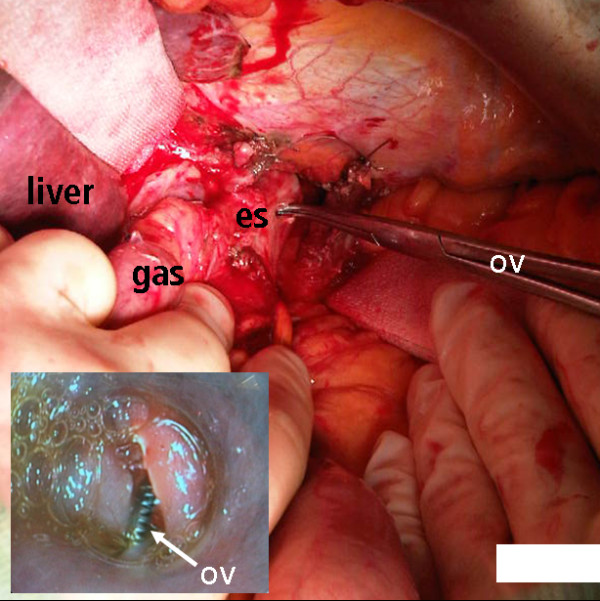
**The small perforation on the left side of the esophagus (es) was in a typical location only 2 cm above the esophageogastric border and was intubatable with the tip of an Overholt clamp (ov) (gas, stomach; es, esophagus)**.

**Figure 6:**
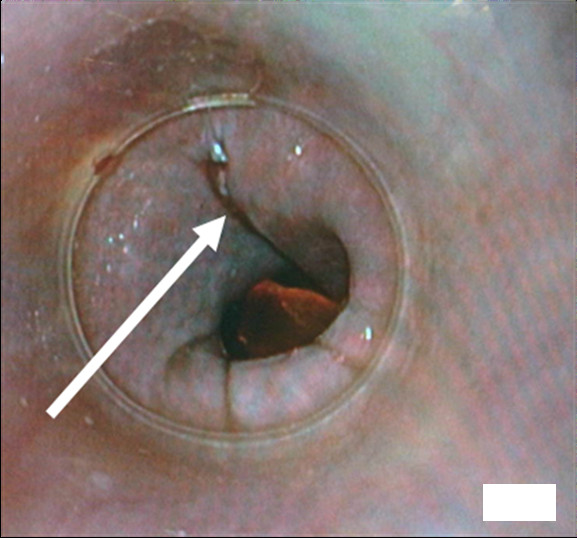
**Intra-operative endoscopic view of the repair result**. Arrow, site of rupture.

During surgery, a small perforation of 3 mm in diameter was found just 2 cm above the cardia on the left side of the esophagus (Figure 5). The intra-operative endoscopy verified the perforation at the location where it had been suspected earlier (ov, Overholt clamp). The lesion was repaired with five stitches of PDS 3-0 suture and Nissen fundoplication. The result of the repair was examined by control endoscopy (Figure 6). During the post-operative course the CT-guided application of an additional pigtail drain for persistent left thoracic abscess formation was necessary. Other than that, her post-operative course was uneventful and our patient recovered completely. She was discharged from hospital three weeks after the initial incident.

## Discussion

Due to its rare incidence, most Boerhaave reports in the medical literature are case reports, (for example, [14-20]), and rarely a larger series of patients with Boerhaave from a single center [9]. For Boerhaave syndrome, excessive vomiting is an absolute prerequisite, and this was also true in our patient's case. But, while excessive vomiting in almost all other cases was spontaneous and, except for one reported case [13], was independent of any elective iatrogenic intervention, in our patient's case vomiting was triggered by a routine and very common procedure of colonoscopy preparation.

Diagnostic investigations and treatment of our patient were not spectacular; however, as our patient presented with the typical signs of persistent and slightly increasing back pain, which started immediately after vomiting, diagnostic investigations by esophagogastroscopy and CT scan with oral CM easily revealed a Boerhaave perforation at the esophagogastric junction.

The initial idea to use vacuum endo-sponge therapy to treat the perforation arose because we have been using this kind of interventional therapy successfully for the treatment of anastomotic insufficiencies in upper gastrointestinal surgery [21,22]. However in this case, and perhaps due to the small size of the perforation, the vacuum seemed to have no draining effect on the gradually forming mediastinal abscess, and consequently the condition of our patient slowly deteriorated. For this reason, we decided to stop the endo-sponge VAC therapy and treat her by open surgery with direct suture and covering by Nissen fundoplication.

Our patient later recalled that she had a history of becoming nauseous easily, but this information was never documented, and nor did she pass on this information at the time of the colonoscopy clarification interview. It is also likely that possible nauseous conditions were not addressed by the interview at all. However, since an anti-emetic medication might have prevented this unfortunate event, it is important to pay attention to possible nauseous conditions before a planned colonoscopy preparation.

## Conclusions

In view of the risk of a severe complication connected with an elective colonoscopy, we conclude that it is justified to prescribe an anti-emetic co-medication as a non-invasive, inexpensive yet effective precaution against excessive vomiting for any routine colonoscopy preparation.

## Consent

Written informed consent was obtained from the patient for publication of this case report and any accompanying images. A copy of the written consent is available for review by the Editor-in-Chief of this journal.

## Competing interests

The authors declare that they have no competing interests.

## Authors' contributions

NE and JK had the idea of reporting this case. NE was in charge of our patient, and diagnosed and treated our patient. He collected the data, analyzed the case, developed the concept of the manuscript and composed it. JK, MDJ and MW provided major writing assistance. All authors read and approved the final manuscript.

## References

[B1] Derbes VJ Mitchell RE Hermann Boerhaave's Atrocis, nec descripti prius morbid historia. The first translation of the classic report of rupture of the esophagus with annotations Bull Med Libr Assoc 1955 43 217 239 14364044 PMC199852

[B2] Weiss S Mallory GK Lesions of the cardiac orifice of the stomach produced by vomiting JAMA 1932 16 1353 1355

[B3] Younes Z Johnson DA The spectrum of spontaneous and iatrogenic esophageal injury: perforations, Mallory-Weiss tears, and hematomas J Clin Gastroenterol 1999 29 306 317 10.1097/00004836-199912000-00003 10599632

[B4] Kovacs TOG Jensen DM Endoscopic diagnosis and treatment of bleeding Mallory-Weiss tears Gastrointest Endosc Clin North Am 1991 1 387 400

[B5] Korn O Onate JC Lopez R Anatomy of the Boerhaave syndrome Surgery 2007 141 222 228 10.1016/j.surg.2006.06.034 17263979

[B6] Cappell MS Sciales C Biempica L Esophageal perforation at a Barrett's ulcer J Clin Gastroenterol 1989 11 663 666 10.1097/00004836-198912000-00013 2584667

[B7] Curci JJ Horman MJ Boerhaave's syndrome: the importance of early diagnosis and treatment Ann Surg 1976 183 401 408 10.1097/00000658-197604000-00013 1267496 PMC1344212

[B8] Plein K Kellner C Hotz J Boerhaave syndrome. The differential diagnosis of acute retrosternal pain [in German] Dtsch Med Wochenschr 1989 114 1153 1156 10.1055/s-2008-1066733 2752920

[B9] Patton AS Lawson DW Shannon JM Risley TS Bixby FE Reevaluation of the Boerhaave syndrome. A review of fourteen cases Am J Surg 1979 137 560 565 10.1016/0002-9610(79)90131-4 426207

[B10] Heberer G Laushke H Hau T Pathogenesis, clinical features and therapy of esophageal ruptures [in German] Chirurg 1966 37 433 440 5928969

[B11] Meyers J Über die Zerreissung der Speiseröhre Preussische Medicinalzeitung (Berlin) 1859 30 29

[B12] Dagash HI Baillie C Lawson RA Will AM Boerhaave syndrome following chemotherapy in a child with acute lymphoblastic leukemia Pediatr Blood Cancer 2004 43 91 92 10.1002/pbc.20042 15170899

[B13] Puschel K An unusual case of Boerhave syndrome. Esophageal rupture during preparation for gastroscopy [in German] Dtsch Med Wochenschr 1985 110 726 728 10.1055/s-2008-1068895 3922725

[B14] Korczynski P Krenke R Fangrat A Kupis W Orlowski TM Chazan R Acute respiratory failure in a patient with spontaneous esophageal rupture (Boerhaave syndrome) Respir Care 2011 56 347 350 10.4187/respcare.00900 21255506

[B15] Paluszkiewicz P Bartosinski J Rajewska-Durda K Krupinska-Paluszkiewicz K Cardiac arrest caused by tension pneumomediastinum in a Boerhaave syndrome patient Ann Thorac Surg 2009 87 1257 1258 10.1016/j.athoracsur.2008.08.009 19324162

[B16] Ng CS Mui WL Yim AP Barogenic esophageal rupture: Boerhaave syndrome Can J Surg 2006 49 438 439 17234078 PMC3207543

[B17] Marshall WB Boerhaave syndrome: a case report AANA J 2002 70 289 292 12242927

[B18] Level C de Precigout V Lasseur C Hachem D Berge F Larroumet N Carles J Blanchetier V Videau J Combe C Aparicio M Spontaneous rupture of the esophagus (Boerhaave syndrome) in a patient with scleroderma treated by continuous ambulatory peritoneal dialysis [in French] Rev Med Interne 1997 18 566 570 10.1016/S0248-8663(97)80809-0 9255375

[B19] Grazioli S Olivetti L Bergonzini R Matei M Capra S Boerhaave syndrome: report of 2 cases [in Italian] Radiol Med 1997 93 306 308 9221432

[B20] Larrieu AJ Kieffer R Boerhaave syndrome: report of a case treated non-operatively Ann Surg 1975 181 452 454 10.1097/00000658-197504000-00015 1130863 PMC1343787

[B21] Wedemeyer J Brangewitz M Kubicka S Jackobs S Winkler M Neipp M Klempnauer J Manns MP Schneider AS Management of major postsurgical gastroesophageal intrathoracic leaks with an endoscopic vacuum-assisted closure system Gastrointest Endosc 2010 71 382 386 10.1016/j.gie.2009.07.011 19879566

[B22] Wedemeyer J Schneider A Manns MP Jackobs S Endoscopic vacuum-assisted closure of upper intestinal anastomotic leaks Gastrointest Endosc 2008 67 708 711 10.1016/j.gie.2007.10.064 18374029

